# Characteristics of Trees Infested by the Invasive Primary Wood-Borer *Aromia bungii* (Coleoptera: Cerambycidae)

**DOI:** 10.3390/insects13010054

**Published:** 2022-01-04

**Authors:** Yuichi Yamamoto, Yosuke Ishikawa, Kazuhiko Uehara

**Affiliations:** 1 Research Institute of Environment, Agriculture and Fisheries, Osaka Prefecture, 442, Shakudo, Habikino, Osaka 583-0862, Japan; 2 Kobe Plant Protection Station, 1-1, Hatoba-cho, Chuo-ku, Kobe, Hyogo 650-0042, Japan; yosuke_ishikawa310@maff.go.jp; 3 Biodiversity Center, Research Institute of Environment, Agriculture and Fisheries, Osaka Prefecture, 10-4, Koyamoto-machi, Neyagawa, Osaka 572-0088, Japan; uehara@mbox.kannousuiken-osaka.or.jp

**Keywords:** *Aromia bungii*, bark roughness, Cerambycidae, host preference, invasive species, red-necked longhorn beetle, Rosaceae, wood-borer, tree characteristics, tree vigor

## Abstract

**Simple Summary:**

The red-necked longhorn beetle *Aromia bungii* is an invasive species that causes damage to Rosaceae trees. In introduced countries, tree damage by *A. bungii* in many regions, such as orchards, forests, and ornamentals, is a serious problem. Information about the characteristics of pest-infested host trees is helpful for efficiently finding and controlling this beetle. In this study, we investigated the characteristics of infested and uninfested ornamental cherry trees in the field, and speculated as to the traits that are important to infestation. As a result, host trees with rough surface bark, large in size, and weakened conditions are prone to damage by *A. bungii*. These findings aid in the selection of trees or locations to be surveyed for intrusion detection surveillance, where a high probability of damage can be found, among many candidates in intruded areas. Moreover, this knowledge is useful for prioritizing preventive measures for host trees that are more likely to be attacked in already invaded areas.

**Abstract:**

The expanding distribution and tree damage of the invasive, primary wood-borer *Aromia bungii* (Coleoptera: Cerambycidae), which kills trees of the Rosaceae family, is a problem in intruded areas. However, the tree characteristics associated with infestation by *A. bungii*, which are useful for early detection or prioritizing preventive measures, are not well examined. We investigated the presence or absence of tree damage (response variable) in pre- and post- surveys along with tree characteristics (four explanatory variables; bark roughness, size, species, and vigor) on monitoring trees in uninvaded sites (survey for the first trees to be damaged) and already invaded sites (survey for the next trees to be damaged). We evaluated the variables using generalized linear mixed models for each site (i.e., a first trees model and a next trees model). Three tree characteristics (bark roughness, size, and vigor) were included as explanatory variables in both best models, indicating that trees with rough surface bark, large in size, and weakened conditions were more susceptible to *A. bungii* infestation. The reasons for the difference between the two models (species was only chosen in the next trees model) will be considered in our future work.

## 1. Introduction

The red-necked longhorn beetle *Aromia bungii* (Faldermann, 1835; Coleoptera: Cerambycidae) is an invasive, primary wood-borer that damages trees of the Rosaceae family [1]. Its main hosts are *Prunus armeniaca* L. and *Prunus persica* (L.) Batsch in original regions [1]. In recent years, damage of trees, including other hosts such as *Prunus domestica* L., *Prunus salicina* Lindl., *Prunus mume* Siebold & Zucc., and flowering cherry trees (*Cerasus* trees), by *A. bungii* in orchards, forests, and ornamental trees is a serious problem in the affected countries: Germany, Italy, and Japan [1,2,3,4].

To prevent the expansion of the distribution of exotic species and tree damage in introduced areas, early detection is critical [5]. A usual method for finding targets is monitoring surveys for intrusion detection based on the presence or absence of the species near the area where it was first discovered. In such surveillance programs, the use of synthetic pheromone lures, in combination with a trap apparatus to capture the beetles, have been conventionally employed against invaders, including exotic cerambycids beetles [6,7]. In addition, an attractant and sex-aggregating pheromone has been identified and artificially synthesized in *A. bungii* [8]. Moreover, studies on sex-specific chemoreceptivity on adult antennae [9] to volatile organic compounds from trees [10] that could be viable candidates for pheromone lure blends were conducted. However, the practical application of pheromone traps for the reconnaissance of adults remains a work in progress [11,12]. Therefore, adopting a fixed-point survey at potential points-of-entry [6] is currently one of the best means for early detection. When selecting suitable hosts and/or sites for an *A. bungii* invasion monitoring survey, information concerning the tree characteristics related to infestation can be helpful.

In relation to tree-boring cerambycid beetle infestations, previous studies have focused on tree characteristics, such as tree size [13,14,15], tree species [16], tree vigor [17,18,19], bark roughness [20,21], and micro-climate [22]. Recent observational reports have suggested that host trees that are larger in size [23,24,25], more mature [3], more stressed [3], and with rolled up or cracked bark [26] are more prone to being attacked by *A. bungii*; however, the factor and/or combinations of factors that decisively affect infestation in the field are unclear. Moreover, previous research on *A. bungii* have referred to tree conditions after damage by *A. bungii* having been found; in these studies, there is no denying the possibility that the condition of the infested trees was a result of the damage caused by the beetles [2]. Therefore, an early survey of tree characteristics is required before the effects of infestation become visible.

This study aimed to clarify the tree characteristics related to *A. bungii* infestation using ornamental cherry trees as monitoring trees in the field. We conducted a pre-survey for infestation, a survey for assessing tree characteristics (four variables: bark roughness, species, tree size, and tree vigor), and a post-survey for the infestation of monitoring trees planted in the field study sites (public parks). Then, we analyzed the characteristics of trees that were damaged and undamaged by *A. bungii* using generalized linear mixed models (GLMMs). We used two types of study site for this analysis, hence we conducted two GLMMs: (1) uninvaded sites for the model of the first trees to be damaged; and (2) already invaded sites for the model of the next trees to be damaged. Knowledge of the characteristics of the first trees to be damaged will aid in the selection of monitoring trees and sites for *A. bungii* intrusion detection. In addition, information about the characteristics of the next trees to be damaged may be useful in prioritizing the application of preventive control measures in intruded areas. Based on our results, we discuss the selections of each factor in the GLMMs from the view point of insect searching behavior, as well as the cause for differences between the models from the stand point of an invasion process related to propagule pressure.

## 2. Materials and Methods

### 2.1. Study Sites and Monitoring Trees

**Figure 1:**
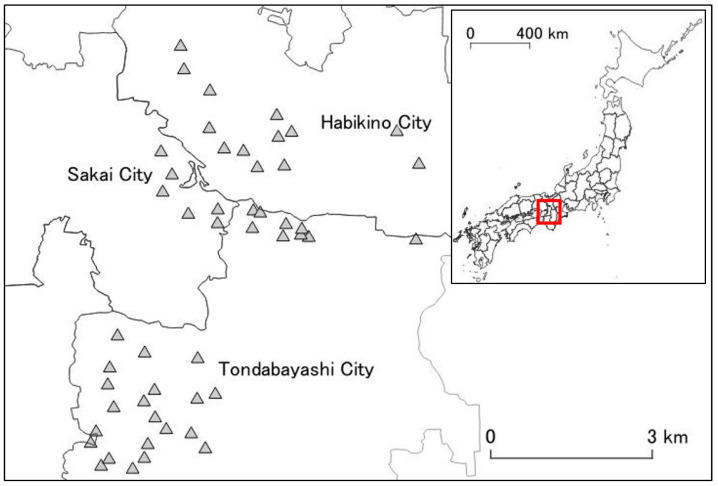
Research area and 49 study sites in Osaka Prefecture. The area spanned three cities in Osaka Prefecture (Habikino City, Sakai City, and Tondabayashi City). The shaded triangles represent the study sites. The map in the top right corner is a map of Japan’s prefectures. Red rectangle represents the location of the research area (Osaka Prefecture).

The research area was set in a region where an initial invasion of *A. bungii* was estimated to have occurred in Osaka Prefecture [27]. The area consisted of both industrial and residential areas. The host trees were planted in areas such as public parks, streets, personal residential properties; in these regions, the host trees were mainly ornamental cherry trees (*Cerasus* spp.), and small orchards (mainly *P. mume*) [24]. In our study, 49 public parks in the area were chosen as study sites; these parks each had four or more ornamental cherry trees (Figure 1).

All the ornamental cherry trees in each study site were established as monitoring trees. In 6 out of 49 survey points, where many flowering cherry trees had been planted, more than 25 trees that had been planted adjacent to each other were arbitrarily selected as monitoring trees.

### 2.2. Survey of Infestation and Tree Characteristics

In general, *A. bungii* has a 2-year life cycle in Japan and adults of *A. bungii* occur from June to August [2]. The larvae of a new generation, hatched from eggs laid by adults, will start to develop inside the host tree. During the hatching year, it is usually difficult to identify the infestation via larval frass ejection [28], which has been used as an indicator of infestation in field studies [24,25,27], because annual larvae discharge tunneling frass at an undetectably small size and low volume. After the first overwintering inside the trees, *A. bungii* larvae resume feeding on the hosts and ejecting more visible frass. Therefore, the ejected frass noted during the adult emergence period (i.e., from June to August) is most likely due to the larvae born in the previous year.

Firstly, from July to August 2017, the initial survey for *A. bungii* infestation (hereafter referred to as the pre-survey) was conducted on each monitoring tree at all study sites, using the presence or absence of frass ejection as an indicator of infestation [27]. Other Cerambycidae species, such as *Anoplophora chinensis* (Forster) (Coleoptera: Cerambycidae) and *Aegosoma sinicum* White (Coleoptera: Cerambycidae), also infest living flowering cherry trees with larval frass emission [29]. Infestation by *A. bungii* larvae was identified based on the particle shape of the frass [29,30,31] ejected from the monitoring trees.

**Figure 2:**
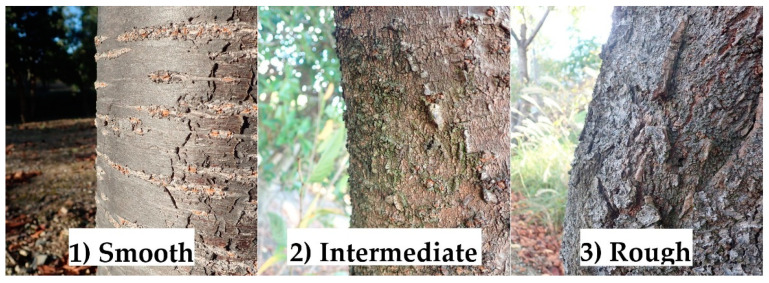
Three categories of tree trunk bark roughness based on the degree of uneveness and cracking of the surface bark, as assessed via visual inspection. All pictures are of *Cerasus × yedoensis* ‘Somei-yoshino’.

Secondly, from September to October 2017, three characteristics were noted in the monitoring trees in the same study location: (1) bark roughness, (2) tree size, and (3) tree vigor. Bark roughness on tree trunks at a height of less than 2 m from the ground, where most ornamental cherry trees were damaged [24], was classified into three categories based on the degree of unevenness and cracking of the surface bark, according to visual inspection: (1) smooth, (2) intermediate, or (3) rough (Figure 2). Tree size was measured as the diameter (cm) of tree trunks at about 10 cm in height from the ground. Tree vigor was rated visually and categorized into five levels based on the attachment of leaves in the crown, with reference to the rating by Hishinuma et al. [32]: level 1, about 75% and more leaf area loss; level 2, about 50% loss; level 3, about 25% loss; level 4, about 10% loss (no tip leaves compared to level 5); and level 5, no loss (fully covered with leaves to tips). Additionally, trees with no crown leaves were treated as dead, classified as level 0 for convenience, and excluded from the analyses.

Thirdly, in April 2018, the species, races, or cultivars (hereafter referred to as a species) of all monitoring trees, as the fourth tree characteristic, were identified during the cherry blossom season (mainly April in Japan) in all study sites. Species categorization was based on the morphology of both the flowers and leaves of the monitoring trees with main reference to the picture book by Oohara [33]. When classification was not possible using the two morphological indexes, the tree species were categorized as a cultivar group named the ‘*C. Sato-zakura* group’ [34].

Lastly, in August 2018, a resurvey for *A. bungii* infestation (hereafter referred to as the post-survey) was performed on the monitoring trees at all study sites, one year after the pre-survey, using the same methods [27].

### 2.3. Trees of Interest by Study Site Category

Table 1 shows the categories of study sites in terms of the presence or absence of *A. bungii* larvae frass ejection on the monitoring trees during the pre- and/or post-survey. Category 1 is a combination of the absence of larval frass ejection in the pre-survey and presence in the post-survey (Table 1). Similarly, Category 2 is the combination of “absence” and “absence”, Category 3 is that of “presence” and “presence”, and Category 4 is that of “presence” and “absence” in the pre- and post-surveys, respectively (Table 1). Categories 1 and 2 corresponded to parts of uninvaded areas; each monitoring tree in Category 1 was used for an analysis of the first trees to be damaged in uninvaded areas (Table 1). Categories 3 and 4 corresponded to parts of already invaded areas; each monitoring tree in Category 3 was used for an analysis of the next trees to be damaged in already invaded areas (Table 1). The “absence” study sites of both Categories 2 and 4 in the post-survey were not included in this study because we could not assess whether *A. bungii* adults had invaded those study sites in the year of the pre-survey.

### 2.4. Data Processing for Statistical Analysis

In terms of tree vigor, trees in the crown rating level 1 were integrated into level 2 since the sample size of level 1 was small. In terms of species, trees categorized as any species other than the top three most frequent classifications were included into the *C.* Sato-zakura group; thus, the monitoring trees were grouped into four species; “*C. jamasakura*,” “*C.* Sato-zakura group,” “*C. speciosa*,” and “*C. × yedoensis* ’Somei-yoshino’” (Appendix A and B). Four trees in study site category 1 and the nine trees in study site category 3 were dead (tree vigor: level 0) or cut prior to the post-survey; these trees were excluded from the statistical analysis (Table 1).

### 2.5. Statistical Analysis

All statistical analyses were performed in R version 4.0.3 [35] using the packages “car,” “emmeans,” “lme4,” “MASS,” “mgcv,” “multcomp,” and “MuMIn.” Both “ggeffect” and “ggplot2” were used to plot the data.

To examine the tree characteristics of both the first (category 1) and next (category 3) trees to be damaged (i.e., in separate models, and hereafter referred to as the first trees model and the next trees model, respectively), we applied a generalized linear mixed model (GLMM: “glmer” function of the “lme4” package) with a log-link function to explain the presence or absence of *A. bungii* larvae frass ejection on the monitoring trees, as an indicator of infestation (response variable). The probability distribution of the response variable was assumed to be a binomial distribution. The explanatory variables in the full model were as follows: tree size (continuous variable), bark roughness (three levels as ordered categorical variables; smooth < intermediate < rough), tree vigor (four levels as ordered categorical variables; crown rating level 2 < level 3 < level 4 < level 5), species (four groups as a categorical variable: “*C. jamasakura*,” “*C.* Sato-zakura group,” “*C. speciosa*,” and “*C. × yedoensis* ‘Somei-yoshino’”). Note that there was a positive polyserial correlation between tree size and bark roughness in the first trees model (ρ = 0.54) and the next trees model (ρ = 0.51; “polyserial” function of the “polycor” package); we included both variables in the full models and checked the multicollinearity of these variables in the selected models (GLMMs) using the variance inflation factor (VIF; “check collinearity” function of the “performance” package).

First, we conducted a preliminary analysis for the selection of random effect terms based on the full model using the restricted maximum likelihood method, considering “tree size” and “study site” with the following random variable: (1) random intercept (study site), (2) random slope (tree size by study site), (3) independent random intercept and slope, and (4) correlated random intercept and slope. The model with the smallest Akaike’s information criterion (AIC) value [36] was selected as the most predictive model (“AIC” function of the “stats” package). The results of the model selection for random effects are shown up to the models with the second smallest AIC value (e.g., Table 2).

Next, we performed model selection for explanatory variables on the full model, with the random effect selected from the above preliminary analysis using maximum likelihood methods. The most predictive model was chosen among the models with all combinations of the explanatory variables (“dredge” function of “MuMIn” package) according to the smallest AIC value. The results of the model selection for explanatory variables, with the coefficients of continuous variables, are shown up to the models with the second lowest AIC value (e.g., Table 3). In terms of the fixed effects in the best model, the Wald’s II Chi-squared test was conducted (“ANOVA” function of the “car” package) for Chi-squared values and *p*-values were used to analyze the effect of each variable on the response variable. In addition, an ANOVA test was performed on the fixed effects to calculate the mean square for variance of the explanatory variables in the models.

Lastly, we estimated the marginal mean ± 1 standard error (SE) for the fixed effects of the categorical variables in the best model, on a log odds ratio scale (“emmeans” function of the “emmeans” package). Then, we performed a multiple comparison on the difference of the estimated marginal means (EMMs) between the levels within each fixed effect using the Tukey-Kramer honestly significant difference (HSD) test (*p* < 0.05); “pairs” function applied to EMMs) and the results were plotted on a response scale (probability) using values back-transformed from the log odds ratio scale.

## 3. Results

### 3.1. Number of Study Sites and Monitoring Trees Categorized by A. bungii Infestation

Table 1 shows the total number of study sites and monitoring trees with or without infestation in the pre- and post-surveys, according to the study site category. The infested or uninfested monitoring trees in the post-survey were treated as the presence or absence data (response variable) for infestation in the first trees model (GLMM; 85 infested trees and 304 uninfested trees in the Category 1 study sites) and in the next trees model (GLMM; 105 infested trees and 171 uninfested trees in the Category 3 study sites; Table 1).

The total number of monitoring trees with or without infestation in the pre- and post-survey is presented according to tree characteristics (after data processing) in Appendix A and according to species (prior to data processing) in Appendix B.

### 3.2. First Trees Model

**Figure 3:**
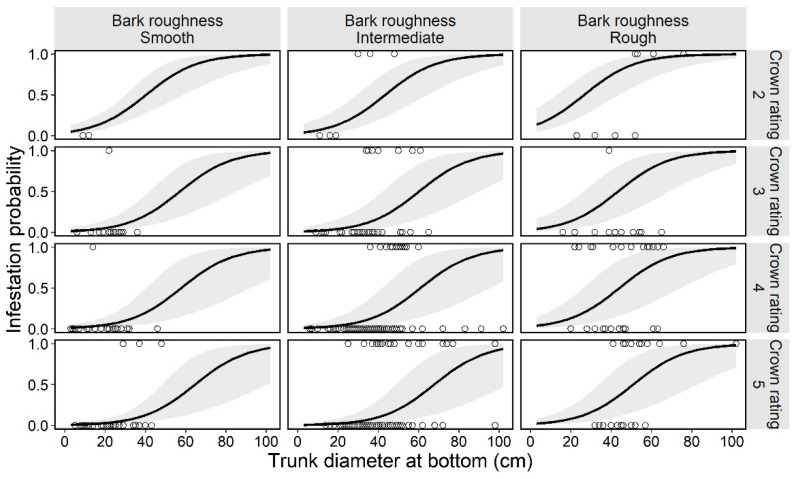
Estimated curve (GLMM) of the probability of an ornamental cherry tree being infested by *Aromia bungii*, in combination with two tree characteristics: bark roughness and tree vigor (crown rating; more vigorous with the numbers) in uninvaded areas. Open circles reflect the recorded value of a monitoring tree for infestation (1) or no infestation (0) in the infestation survey. The shaded area represents the 95% confidence intervals of the mean probability of infestation, without considering the influences of the random effect (random slope for tree size by study site) on the GLMM.

**Figure 4:**
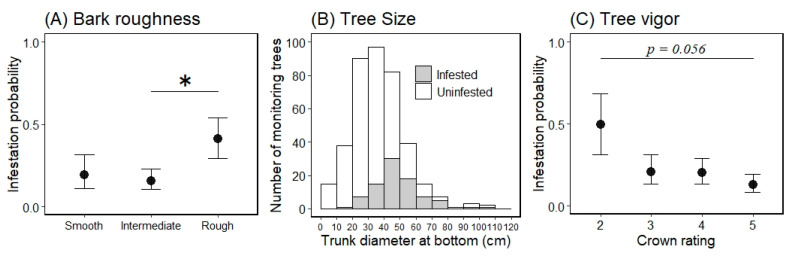
Marginal mean for the probability of an ornamental cherry tree being infested by *Aromia bungii* based on bark roughness (**A**) and tree vigor (**C**), and a histogram of the continuous variable tree size (**B**) for infestation (1) or no infestation (0) in uninvaded areas. Filled circles represent mean values and the bars extending from the circles represent the standard error (SE) of the means (**A**,**C**; back-transformed from the log odds ratio scale for the mean ± 1 SE). Shaded areas represent infested trees and open areas represent uninfested trees in the infestation survey (**B**). * indicates a significant difference in the mean probability of infestation between the levels of the indicated bars (Tukey–Kramer HSD test: *p* < 0.05).

In the preliminary analysis for the selection of random effects in the first trees model, we selected the random slope model for tree sizes by study site (Table 2). Next, the best model (GLMM) for tree infestation, selected according to the AIC value, included the following explanatory variables: bark roughness, tree size, and tree vigor. These three variables (bark roughness, tree size, and tree vigor) did not have multicollinearity (VIF = 1.13, 1.17, and 1.12, respectively). The estimated curve and recorded value by combinations of each level on the fixed effects in the first trees model are shown in Figure 3. The partial regression coefficient for the variable tree size was positive (coefficient = 0.079) in the best model (Table 3), indicating that larger ornamental cherry trees are more susceptible to *A. bungii* infestation, as shown in Figure 4. The mean trunk circumference was 48.6 ± 14.9 cm in infested trees and 33.4 ± 15.2 cm in uninfested trees (36.7 ± 16.4 cm across all monitoring trees). The smallest and largest trunk circumferences at the bottom of infested trees were 14 and 102 cm, respectively. The ordered, categorical variable bark roughness had a positive effect on infestation in the order of the levels (Figure 3). On the other hand, the other ordered, categorical variable tree vigor had a negative effect on infestation in the order of the levels (Figure 3). The results of the parametric coefficients for the categorical variables in the GLMM are shown in Table 3 and Appendix C. The mean squares for variance and Chi-square values of each fixed effect, based on the best model, are shown in Appendix D.

The marginal means ± 1 SEs of each level for the variable bark roughness on a log odds ratio scale were −1.441 ± 0.670 for the smooth level, −1.687 ± 0.467 for the intermediate level, and −0.362 ± 0.521 for the rough level (Figure 4: back-transformed from the log odds ratio scale for the mean ± 1 SE). When comparing the marginal mean probability of infestation between the bark roughness levels, we only found a significant difference between the intermediate and rough levels (Tukey HSD test, difference in the means = 1.325, *p* < 0.05); there were no other differences between any other levels (*p* > 0.05; Figure 4). The marginal means for tree vigor, on a log odds ratio scale, were −0.010 ± 0.786 for level 2, −1.335 ± 0.552 for level 3, −1.387 ± 0.485 for level 4, and −1.922 ± 0.488 for level 5 (Figure 4: back-transformed from the log odds ratio scale for the mean ± 1 SE). When comparing the marginal mean probability of infestation between tree vigor levels, there were no differences between all levels (Tukey HSD test, *p* > 0.05); however, the value of the lowest (level 2) and highest levels (level 5) tended to be different (Tukey HSD test, difference in the means = 1.913, *p* = 0.056).

### 3.3. Next Trees Model

**Figure 5:**
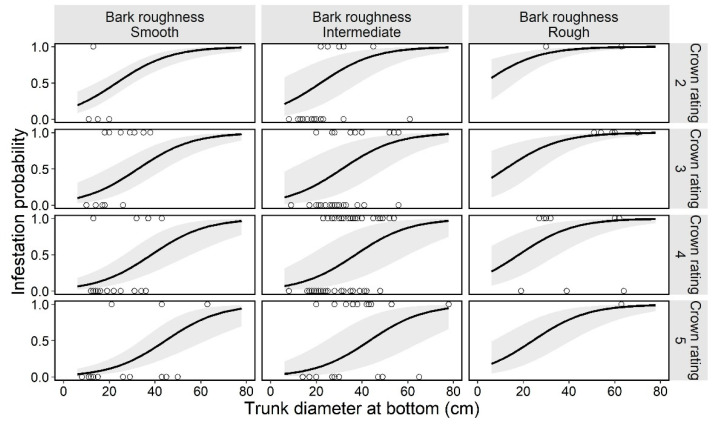
Estimated curve (GLMM) of the probability of an ornamental cherry tree being infested by *Aromia bungii,* in combinations with two tree characteristics: bark roughness and tree vigor (crown rating; more vigorous with the numbers) in *Cerasus* × *yedoensis* ‘Somei-yoshino’ in already invaded areas. Open circles represent the recorded value of a monitoring tree for infestation (1) or no infestation (0) in the infestation survey. Shaded areas represent the 95% confidence intervals of mean probability of infestation, without considering the influences of the random effect (random slope for tree size by study site) on the GLMM.

In the preliminary analysis for the selection of random effects in the next trees model, we selected the random slope model for tree size by study site (Table 4). Next, the best model (GLMM) for tree infestation, selected according to the AIC value, included the following explanatory variables: bark roughness, species, tree size, and tree vigor (Table 5). These four variables (bark roughness, species, tree size, and tree vigor) did not have multicollinearity (VIF = 1.20, 1.11, 1.30, and 1.33, respectively). The estimated curve and recorded value by combinations of each level on the fixed effects are shown for *C. × yedoensis* ‘Somei-yoshino’ as it is representative of most planted species in the study sites (Appendix B) in Figure 5. The partial regression coefficient for the variable tree size was positive (coefficient = 0.08; Table 5), indicating that larger ornamental cherry trees are more susceptible to *A. bungii* infestations, like in the first trees model. The mean trunk circumference was 37.2 ± 13.6 cm in infested trees and 25.9 ± 11.8 cm in uninfested trees (30.2 ± 13.6 cm across all the monitoring trees). The smallest and largest trunk circumferences at the bottom of infested trees were 13 and 78 cm, respectively. The ordered, categorical variable bark roughness had a positive effect on infestation in the order of the levels (Figure 5). On the other hand, the ordered, categorical variable tree vigor had a negative effect on infestation in the order of the levels. The results of parametric coefficients for the categorical variables in the GLMM are shown in Table 5 and Appendix E. The mean squares for variance and Chi-square values of each fixed effect, based on the best model, are shown in Appendix F.

**Figure 6:**
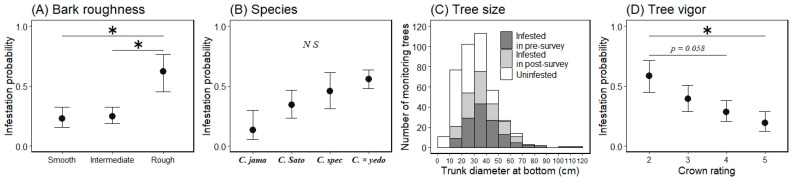
Marginal mean of monitoring trees being infested by *Aromia bungii* based on bark roughness (**A**), species (**B**), and tree vigor (**D**), and a histogram of the continuous variable tree size (**C**) for infestation (1) or no infestation (0) in invaded areas. Filled circles represent mean values and bars extending from the circles represent the standard errors (SEs) of the means (**A**,**B**,**D**; back-transformed from the log odds ratio scale for the mean ± 1 SE). *C. jama*, *C. Sato*, *C. spec*, and *C. × yedo* are abbreviations for *Cerasus jamasakura*, *C.* Sato-zakura group (including all species other than these three species), *C. speciosa*, and *C. × yedoensis* ‘Somei-yoshino’, respectively. Dark shaded areas represent infested trees observed in the pre-survey for infestation, light shaded areas represent infested trees, and open areas represent uninfested trees in the post-survey for infestation (**C**). * indicates a significant difference in the mean probability of infestation between the levels of the indicated bars (*p* < 0.05). *N S* indicates no significant difference between the means of each level (Tukey–Kramer HSD test: *p* > 0.05).

The marginal means ± 1 SEs of each level for bark roughness on a log odds ratio scale were −1.271 ± 0.479 for the smooth level, −1.110 ± 0.369 for the intermediate level, and 0.488 ± 0.673 for the rough level (Figure 6: back-transformed from the log odds ratio scale for the mean ± 1 SE). When comparing the marginal mean probability of infestation between the bark roughness levels, values for the smooth and rough levels were significantly different (Tukey HSD test, difference in the means = 1.704, *p* < 0.05), as were those for the intermediate and rough levels (difference in the means = 1.598, *p* < 0.05); however, the smooth and intermediate levels were the same (*p* > 0.05; Figure 6). The marginal means for species, on a log odds ratio scale, were −1.862 ± 1.005 for *C. jamasakura*, −0.685 ± 0.522 for the *C. Sato-zakura* group, −0.167 ± 0.631 for *C. speciosa*, and 0.235 ± 0.315 for *C. × yedoensis* ‘Somei-yoshino’ (Figure 6: back-transformed from the log odds ratio scale for the mean ± 1 SE). When comparing the marginal mean probability of infestation between the groups of species, there were no differences between any groups (Tukey HSD test, *p* > 0.05; Figure 6). The marginal means for tree vigor, on a log odds ratio scale, were 0.351 ± 0.562 for level 2, −0.435 ± 0.465 for level 3, −0.926 ± 0.425 for level 4, and −1.441 ± 0.531 for level 5 (Figure 6: back-transformed from the log odds ratio scale for the means ± 1 SEs). When comparing the marginal mean probability of infestation between tree vigor levels, the values for level 2 were different from those for level 5 (Tukey HSD test, difference in the means = 1.792, *p* < 0.05), and all other levels were similar (*p* > 0.05; Figure 6); however, the values of the lowest level (level 2) and the second highest level (level 4) tended to be different (Tukey HSD test, difference in the means = 1.277, *p* = 0.058; Figure 6).

## 4. Discussion

We accounted for the characteristics related to infestation susceptibility of host trees by the invasive, primary wood-borer *A. bungii* using two GLMMs—a model for the first trees to be damaged in uninvaded areas and a model for the next trees to be damaged in already invaded areas—based on the presence or absence of frass ejection on each monitoring tree in field surveys. The two GLMMs had three explanatory variables in common (bark roughness, tree size, and tree vigor), with significant coefficients (except for tree vigor in the first trees model; Appendix D and F), and the same random effect (random slope of tree size by study site; Table 2 and Table 3). Moreover, each explanatory variable showed the same directional influences in both models (Figure 3 and Figure 5). Therefore, these three characteristics can be an important determinant for *A. bungii* infestation in host trees.

We showed that larger trees were more susceptible to infestation. Previous research on other Cerambycidae beetles also reported that infested host trees were larger in size [14,15,37]. In terms of host selections, in the case of *Tetropium fuscum* (Fabricius) (Coleoptera: Cerambycidae), tree size positively affects the landing rate; this relationship is thought to be due to the difference in the chances of adults being passively intercepted on larger versus smaller trees [15]. This passive effect related to tree size is also suggested in the mathematical model for encounter rates in the process of random searching for host trees in tree-boring bark beetles [38]. Conversely, adult females of *Semanotus japonicus* Lacordaire (Coleoptera: Cerambycidae) make inter-tree movements by preferentially selecting the larger trees in a patch, and also exhibit more frequent residence, resulting in more occurrences of adults of the next generation on the trees [13]. As for *A. bungii*, there are no reports about random or selective behavior on host trees in the field. It may also be important to consider the invasion process with respect to host selection. The attraction of cerambycids to host trees is thought to occur by host volatiles on a patch scale, then random landing among host trees within a patch would occur after being introduced [18]. Therefore, it may be better to examine the process—(1) entering a patch and (2) landing or ovipositing on a single-tree in a patch—through which *A. bungii* adults can behave randomly or selectively. In future studies, both the mechanism for the selection of invasion patches among uninvaded patches and the mechanism for the infestation of larger trees within a selected patch should be assessed; identifying these mechanisms would provide useful information for selecting the most appropriate sites and trees for monitoring the invasion of *A. bungii*. As a supplementary note, the effect size of the variable (tree size) varies among study sites, as suggested by the selection of the random slope models for tree size. The SD for tree size on the random slope coefficients in the next trees model (0.022) was slightly smaller than that in the first trees model (0.035; Table 3 and Table 5); this may be partly related to differences in the distribution of tree sizes under attack by *A. bungii* adults because larger trees had already been infested (Figure 6), whereas smaller trees were used as monitoring trees for analysis in the next trees model (the mean ± SD of trunk circumference: 36.7 ± 16.4 cm in the first trees model versus 30.2 ± 13.6 cm in the next trees model).

Our results suggest that trees with rough surface bark are more susceptible to infestation by *A. bungii* (Figure 3 and Figure 5). Considering that adult females oviposit in the crevices of bark [1], trees with smooth surface bark (i.e., few crevices) would not have suitable sites for oviposition, whereas trees with rough surface bark would have suitable sites. Besides, trees with smooth tree bark may avoid beetle attacks by reducing the insect’s ability to grip onto the surfaces of the trees (slippery hypothesis) [39]. *A. bungii* females may have difficulty crawling and/or residing on smooth surface trees while searching for appropriate egg-laying sites. On the other hand, the mean infestation probability was similar for the smooth and intermediate levels, based on our visual assessment of bark (Figure 4 and Figure 6); this lack of a difference may be due to the partial coverage of intermediate monitoring trees with smooth bark, which can significantly reduce the number of total attacks by bark beetles [39]. Hence, entire coverage with a rough surface may increase the risk of infestation by *A. bungii*. Further experiments are required to identify the cut-off point for the ratio of rough surface bark on tree surfaces.

With regards to tree vigor, *A. bungii* can cause damage to trees in various conditions: from obviously healthy trees (crown rating: level 5) to weakened trees (crown rating: level 2; recorded values in Figure 3 and Figure 5). This ecological feature of *A. bungii* as a primary wood-borer may classify it as a highly invasive pest [40]. Moreover, our results suggest that the infestation rate of *A. bungii* increased as the tree vigor decreased (Figure 4 and Figure 6). The high damage rate on weakened trees may suggest that tree vigor is related to some infestation process; weakened trees may be selectively chosen as a landing site by *A. bungii* and/or weakened trees (chosen randomly) may be more susceptible to infestation because of the physiological effect of tree decline. Nonetheless, we were unable to assess the process after landing in this study.

Species was differentially selected for inclusion in the first trees model (not selected) and the next trees model (selected; Table 3 and Table 5). Moreover, in the next trees model, species was chosen as an explanatory variable, which did not have a significant effect on the model (Appendix F) and the mean infestation probability among species categories was not different (Figure 6). There are no reports for differences in *A. bungii* infestations among ornamental cherry trees species in the field. When including host species other than ornamental cherry trees, the damage rate of ornamental cherry trees was much lower than that of peach trees (*P. persica*) in the vicinity of a peach orchard, despite the fact that the ornamental cherry trees were larger than the peach trees [23]. In a study of the invasive tree-boring Cerambycidae beetle *Anoplophora glabripennis* (Motschulsky), the host species strongly affected infestation in both the field and laboratory experiments [41]. There is a lack of clear evidence for differences in the susceptibility to *A. bungii* infestation between host species; therefore, further research is necessary, both in the field and in laboratory studies.

Upon consideration of the values for the conditional coefficient of determination (R^2^) in the best models (Table 3 and Table 5), some factors may explain the residual variations of the models. In the process of the invasion of non-native species, the probability of success for intrusion into new regions increases with both the size of a set of individuals released and the number of release events—this is called propagule pressure [5]. Scaling this process down and applying it to our research scheme, the establishment of *A. bungii* infestation in monitoring trees could also be influenced by propagule pressure. In our study, we assumed that the monitoring trees in the first trees model were under a lower propagule pressure derived from uninvaded sites, whereas the monitoring trees in the next trees model were under a higher propagule pressure derived from the already invaded sites. If so, the difference in both the selection and statistical significance of the variable (e.g., species and tree vigor) may become apparent when propagule pressure varies. In this study, we did not include such indexes for propagule pressure because we were unable to monitor or determine the number and frequency of visits by adult females on monitoring trees. Verification of the models upon inclusion of indexes for propagule pressure is our future challenge.

In summary, using field studies and GLMMs, we showed that the tree characteristics of bark roughness, tree size, and tree vigor could be important indexes for susceptibility to infestation by the primary wood-borer *A. bungii*. Trees with rough surface bark, that are larger in size, and have a weakened condition were more susceptible to infestation. This knowledge aids in the selection of monitoring sites and trees for the detection of *A. bungii* intrusion in undiscovered areas (by the first trees model) and to prioritize trees for the application of preventive control measures in already invaded locations (by the next trees model). Moreover, there are other potential, influencing factors for *A. bungii* infestation, such as host tree species and propagule pressure; our future research will examine the effect of these factors on the infestation process.

## Figures and Tables

**Table 1:** Study site category by presence or absence of a tree infested by *Aromia bungii* and the total number of monitoring trees with and without infestation in each study site category. Pre-survey was the first survey on monitoring trees to classify them as infested or uninfested trees. Post-survey was the second survey on the monitoring trees, which were uninfested in the pre-survey, to categorize them as infested or uninfested trees.

Category of Study Site	Infested Trees in Study Site		Total Number of Study Sites	Number of Trees				Total Number of Monitoring Trees
	In Pre-Survey	In Post-Survey		Infested in Pre-Survey	Infested in Post-Survey	Uninfested in Post-Survey	Cut or Dead before Post-Survey	
1	Absence	Presence	12	0	85	304	4	393
2	Absence	Absence	13	0	0	182	3	185
3	Presence	Presence	22	132	105	171	9	417
4	Presence	Absence	2	14	0	22	1	37

**Table 2:** Results of the preliminary selection of random effect terms in generalized linear mixed models (GLMMs) for tree infestation by *Aromia bungii* in uninvaded areas.

Rank	Random Effect Term	ΔAIC
1	Slope (tree size) by study sites	0
2	Independent intercept and slope (tree size) by study site	0.44

Explanatory variables in the GLMM (full model): bark roughness + species + tree size + tree vigor. AIC, Akaike’s information criterion; GLMM, generalized linear mixed model.

**Table 3:** Results of model selection by AIC and parametric coefficients of GLMMs, based on the preliminary selection in Table 2.

Rank	ΔAIC	R^2^		Regression Coefficient					Standard Deviation of Coefficient
		Conditional	Marginal	Bark Roughness	Species	Tree Size	Tree Vigor	Intercept	Tree Size (Random Slope)
1	0	0.567	0.307	+	−	0.079	+	−4.054	0.035
2	0.65	0.529	0.275	+	−	0.069	−	−4.069	0.033

Plus (+) and minus (−) symbols indicate that the categorical variable was selected and not selected in the model, respectively. AIC, Akaike’s information criterion; GLMM, generalized linear mixed model.

**Table 4:** Results of the preliminary selection of random effect terms on GLMMs for tree infestation by *Aromia bungii* in invaded areas.

Rank	Random Effect Term	ΔAIC
1	Slope (tree size) by study sites	0
2	Independent intercept and slope (tree size) by study sites	2.00

Explanatory variables in the GLMM (full model): bark roughness + species + tree size + tree vigor. AIC, Akaike’s information criterion; GLMM, generalized linear mixed model.

**Table 5:** Results of model selection by AIC and parametric coefficients of GLMMs, based on the preliminary selection in Table 4.

Rank	ΔAIC	R^2^		Regression Coefficient					Standard Deviation of Coefficient
		Conditional	Marginal	Bark Roughness	Species	Tree Size	Tree Vigor	Intercept	Tree Size (Random Slope)
1	0	0.471	0.382	+	+	0.083	+	−2.689	0.022
2	1.83	0.481	0.338	+	−	0.089	+	−2.632	0.029

Plus (+) and minus (−) symbols indicate that the categorical variable was selected and not selected in the model, respectively. AIC, Akaike’s information criterion; GLMM, generalized linear mixed model.

## Data Availability

All data analyzed in this study are included in this article and appendix.

## Appendix A

**Table A1:** Number of monitoring trees for infestation by *Aromia bungii*, according to tree characteristics during the pre-and post-surveys for infestation in each study site.

Tree Characteristic	Level or Class	Number of Monitoring Trees						
		Study Site Category 1			Study Site Category 3			
		Infested in Post- Survey	Uninfested in Post- Survey	Total for Analysis	Infested in Pre- Survey	Infested in Post- Survey	Uninfested in Post- Survey	Total for Analysis
Bark roughness	Smooth	5	70	75	12	17	56	73
	Intermediate	48	194	242	90	67	107	174
	Rough	32	40	72	30	21	8	29
Species	*Cerasus jamasakura*	1	7	8	0	2	12	14
	*C.* Sato-zakura group	1	44	45	15	10	44	54
	*C. speciosa*	8	37	45	4	7	11	18
	*C. × yedoensis* ‘Somei-yoshino’	75	216	291	113	86	104	190
Tree size (cm)	1–10	0	15	15	0	0	11	11
	11–20	1	37	38	9	12	56	77
	21–30	7	83	90	29	25	48	102
	31–40	15	82	97	43	32	38	113
	41–50	30	52	82	27	16	14	57
	51–60	18	21	39	13	13	1	27
	61–70	7	8	15	5	6	3	14
	71–80	5	2	7	2	1	0	3
	81–90	0	1	1	2	0	0	2
	91–100	1	2	3	0	0	0	0
	101–110	1	1	2	1	0	0	1
	111–120	0	0	0	1	0	0	1
Tree vigor (crown rating)	Level 2	7	10	17	27	17	20	37
	Level 3	11	62	73	49	28	45	73
	Level 4	32	112	144	46	42	66	108
	Level 5	35	120	155	10	18	40	58

## Appendix B

**Table A2:** Number of monitoring trees for infestation by *Aromia bungii,* classified by species, varieties, or cultivars (scientific name) during the pre- and post-surveys for infestation in each study site.

Species, Varieties, or Cultivars (Scientific Name)	Study Site Category 1		Study Site Category 3			Total Number of Monitoring Trees in Pre- Survey
	Infested in Post- Survey	Uninfested in Post- Survey	Infested in Pre- Survey	Infested in Post- Survey	Uninfested in Post- Survey	
*Cerasus itosakura* ‘Pendula’	0	5	0	0	1	6
*C. jamasakura*	1	7	0	2	12	22
*C. leveilleana*	0	3	0	0	3	6
*C. leveilleana* ‘Juzukakezakura’	0	1	0	0	0	1
*C. sargentii*	0	2	2	1	4	9
*C.* Sato-zakura Group	0	15	8	7	31	61
*C.* Sato-zakura Group ‘Albo-rosea’	0	8	0	0	0	8
*C.* Sato-zakura Group ‘Beni-yutaka’	0	1	0	0	0	1
*C.* Sato-zakura Group ‘Hisakura’	0	0	0	1	0	1
*C.* Sato-zakura Group ‘Sekiyama’	1	4	0	0	0	5
*C. speciosa*	8	37	4	7	11	67
*C. speciosa* ‘Surugadai-odora’	0	5	0	0	5	10
*C. × yedoensis* ‘Somei-yoshino’	75	216	113	86	104	594
N.A.	0	0	5	1	0	6

## Appendix C

**Table A3:** Regression coefficients of the ordered categorical variables in the prediction model (GLMM) for tree infestation by *Aromia bungii* in uninvaded areas.

Explanatory Variable	Estimate	Std. Error	*z* Value	Pr (>|*z*|)	
Bark roughness.L.	0.763	0.461	1.653	0.098	.
Bark roughness.Q.	0.642	0.326	1.971	0.049	*
Tree vigor.L.	−1.295	0.520	−2.489	0.013	*
Tree vigor.Q.	0.395	0.420	0.939	0.348	
Tree vigor.C.	−0.393	0.352	−1.117	0.264	

Explanatory variables in the GLMM formula: bark roughness + tree size + tree vigor + (0 + tree size | study site). * *p* < 0.05, . *p* < 0.1.

## Appendix D

**Table A4:** Mean squares for variance and Chi-square values of each fixed effect based on the best model (GLMM) for tree infestation by *Aromia bungii* in uninvaded areas.

Fixed Effect	Df	Sum of Squares for Variance	Mean Squares for Variance	Chi-Square Values	Pr (>Chisq)
Bark roughness	2	13.02	6.51	9.44	0.007
Tree size	1	15.18	15.18	19.48	<0.001
Tree vigor	3	6.88	2.29	6.74	0.081

Explanatory variables in the GLMM formula: bark roughness + tree size + tree vigor + (0 + tree size | study site).

## Appendix E

**Table A5:** Regression coefficients of the ordered categorical variables in the prediction model (GLMM) for tree infestation by *Aromia bungii* in invaded areas.

Explanatory Variable	Estimate	Std. Error	*z* Value	Pr (>|*z*|)	
Bark roughness.L.	1.205	0.504	2.392	0.017	*
Bark roughness.Q.	0.609	0.326	1.867	0.062	.
*Cerasus × yedoensis* ‘Somei-yoshino’	0.402	0.618	0.651	0.515	
*C.* Sato-zakura group	−0.490	0.765	−0.641	0.522	
*C.* jamasakura	−1.695	1.146	−1.480	0.139	
Tree vigor.L.	−1.312	0.421	−3.114	0.002	**
Tree vigor.Q.	0.135	0.344	0.394	0.694	
Tree vigor.C.	−0.072	0.296	−0.242	0.809	

Explanatory variables in the GLMM formula: bark roughness + species + tree size + tree vigor + (0 + tree size | study site). ** *p* < 0.01, * *p* < 0.05, . *p* < 0.1.

## Appendix F

**Table A6:** Mean squares for variance and Chi-square values of each fixed effect based on the best model (GLMM) for tree infestation by *Aromia bungii* in invaded areas.

Fixed Effect	Df	Sum of Squares for Variance	Mean Squares for Variance	Chi-Square Values	Pr (>Chisq)
Bark roughness	2	11.82	5.91	6.48	0.039
Species	3	6.21	2.07	6.93	0.074
Tree size	1	16.53	16.53	22.63	<0.001

Explanatory variables in the GLMM formula: bark roughness + tree size + tree vigor + (0 + tree size | study site).
